# Video-assisted thoracoscopic surgery for bilateral intralobar pulmonary sequestration

**DOI:** 10.1016/j.ijscr.2018.10.060

**Published:** 2018-10-29

**Authors:** Norichika Iga, Hideyuki Nishi, Shinichiro Miyoshi

**Affiliations:** Department of Surgery, Okayama Rosai Hospital, 1-10-25 Chikkomidorimachi, Minamiku, Okayama, 702-8055, Japan

**Keywords:** ILS, intralobar pulmonary sequestration, VATS, video-assisted thoracoscopic surgery, CT, computed tomography, Intralobar pulmonary sequestration, Thoracoscopic surgery, Limited resection

## Abstract

**Introduction:**

Intralobar pulmonary sequestration (ILS) is a rare congenital malformation, and is supplied by an aberrant systemic artery. The conventional surgical resection for ILS is lobectomy through an open thoracotomy. However, lobectomy might lead to a significantly compromised pulmonary function. There have been some reports on the feasibility or advantages of the VATS approach in recent years. We report a rare case where we performed a two staged segmentectomy and wedge resection of the affected lesions using a VATS approach for bilateral ILS, and achieved a successful outcome.

**Case presentation:**

A 30-year-old woman was diagnosed with pneumonia. Chest CT showed multiple cystic masses containing fluid, in the posterobasal segment of the right lung and a hyper infiltrated area in the identical segment of the left lung. 3D CT angiography showed that the area of the right sided lesion received its blood supply from the left gastric artery and the area of the left sided lesion from the proximal celiac artery. The patient was diagnosed with bilateral ILS, and a staged, sequential lung resection was planned using the VATS approach. Anatomical basilar segmentectomy with VATS minithoracotomy for the right ILS, and resection of only the sequestrated lung by VATS for the left ILS were performed.

**Conclusions:**

It is important to plan the surgical treatment strategy for bilateral ILS for the preservation of respiratory function. Limited resection using the VATS approach for bilateral ILS can be performed safely and might be useful as a minimally invasive surgery.

## Highlights

• ILS is a rare congenital malformation, and is supplied by an aberrant systemic artery.
• Planning surgical treatment strategy for bilateral ILS is important for preserving respiratory function.
• Limited resection using VATS for bilateral ILS can be safe and useful as minimally invasive surgery.

## 1 Introduction

Intralobar pulmonary sequestration (ILS) is a rare congenital malformation, characterized by a nonfunctional lung segment without a normal tracheobronchial tree, and fed by an aberrant systemic artery [1]. Over the decades, conventional surgical resection for ILS has been lobectomy through open thoracotomy. Although there have been some reports on the feasibility and advantages of the VATS approach in recent years [[2], [3], [4]], lobectomy has been performed for excision of the sequestrated lesion in most cases. However, lobectomy might result in the loss of a large volume of the normal lung. Bilateral lobectomy by open thoracotomy for bilateral ILS might lead to a deterioration in the respiratory function and in the quality of life. We performed a two staged segmentectomy and wedge resection for the affected lesion using a VATS approach for bilateral ILS, with a successful outcome. The work has been reported in line the SCARE criteria [5].

## 2 Presentation of case

A 30-year-old woman, presented with high fever, and pain in the right flank. The chest-X-ray revealed infiltrative shadows in the right lower lung, without any signs of pleural effusion. She was diagnosed with pneumonia and treated with antibiotic therapy.

**Fig. 1:**
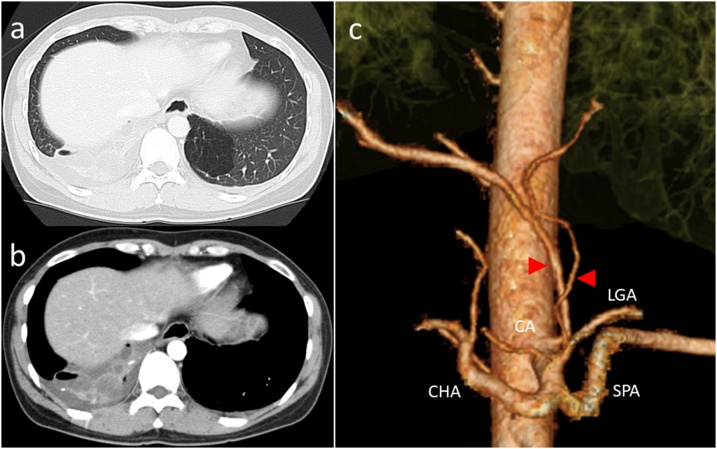
Chest computed tomography (CT) shows bilateral intralobar pulmonary sequestration. CA, Celiac artery; LGA, Left gastric artery; CHA, Common hepatic artery; SPA, Splenic artery; **(a)** Multiple cystic lesions in the right lower lobe were detected in the posterobasal segment of the right lung. **(b)** Hyperinflated lesion can be seen in the identical segment of the left lung. **©** Right aberrant artery arising from the celiac artery and left aberrant artery arising from the left gastric artery entering the affected segments (arrowhead).

Chest computed tomography (CT) showed multiple cystic masses, containing fluid in the posterobasal segment of the right lung and an emphysematous change was detected in the identical segment of the left lung (Fig. 1a and b). Congenital cystic lung diseases such as bronchogenic cyst, lung sequestration, and congenital cystic adenomatoid malformation were suspected.

Three-dimensional CT angiography (3D-CTA) showed that the area of the right sided lesion received its blood supply from the left gastric artery and the area of the left sided lesion from the proximal celiac artery (Fig. 1c). Venous drainage from the sequestrations returned through the ipsilateral inferior pulmonary veins, mainly V10 on each side. The patient was diagnosed with bilateral ILS and a staged, sequential lung resection using the VATS approach was planned.

### 2.1 Right sided ILS

**Fig. 2:**
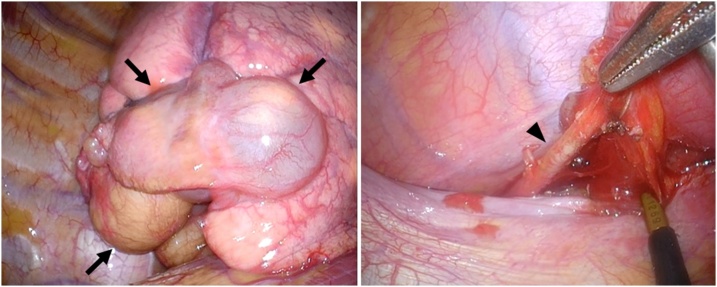
Thoracoscopic findings in the right thoracic cavity. Multiple cystic changes in the right affected segments (arrows). Aberrant artery entered the posterobasal segment through the inferior pulmonary ligament (arrow head).

A-6-cm posterolateral mini thoracotomy was performed over the 5^th^ intercostal space. A 10 mm utility working port was placed at the 5^th^ intercostal space along the anterior axillary line and another 10 mm port for the insertion of a flexible thoracoscope was placed at the 7^th^ intercostal space along the posterior axillary line. Multiple cystic lesions without pleural adhesions were observed in the posterior and lateral basal segment (Fig. 2). The aberrant artery in the inferior pulmonary ligament was isolated and stapled by Endostaplar (Powered ECHELON FLEX® 7). There were no dense adhesions in the hilar structures, and the branches of the normal pulmonary arteries (A9-10) in the affected segments were identified and ligated. The V10 draining the sequestration was divided with ligatures and V9 was divided with the endostapler. Anatomical basilar (S9-10) segmentectomy was performed.

### 2.2 Left sided ILS

**Fig. 3:**
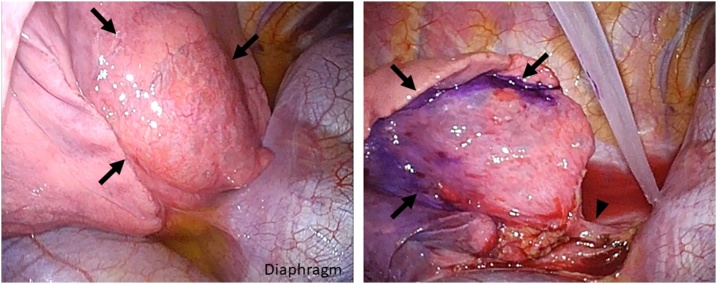
Thoracoscopic findings in the left thoracic cavity. The sequestrated lung is easily identified by the emphysematous change and superficial telangiectasis (arrows). Resection line marked with gentian violet along the demarcated line before ligation of the aberrant artery.

We challenged the sequestrectomy by VATS approach without mini thoracotomy to preserve a better pulmonary function, since the left sided sequestration was not complicated by an infection. A 2.0 cm port for the insertion of a 10 mm flexible thoracoscope and retrieval of the resected lung specimen was made along the anterior axillary line at the 6^th^ intercostal space. A 5 mm utility working port was placed at the 4^th^ intercostal space along the anterior axillary line and a 10 mm port was placed in the 7^th^ intercostal space along the posterior axillary line. A 5 mm retraction port was placed inferior to tip of the scapula. A demarcated line was clearly observed between the sequestrated lung and the normal lung parenchyma, due to the emphysematous change and superficial telangiectasis (Fig. 3). We marked the demarcation line with gentian violet before ligation of the aberrant artery, to avoid confusing it with the resection line once the blood supply would be interrupted. The inferior pulmonary vein was mobilized to avoid involvement in stapling line. The lung parenchyma was dissected with endostaplar, along the marked demarcation line.

She was discharged from the hospital without any complications. At 3-months follow-up, the CT demonstrated no residual sequestration and the normal lung parenchyma was fully preserved.

## 3 Discussion

Pulmonary sequestration is a congenital pulmonary malformation separated from the normal tracheobronchial tree and receives a systemic arterial blood supply. It is a rare developmental abnormality accounting for 0.1 to 6.4% of all congenital lung anomalies [6,7].

Pulmonary sequestration is classified as extralobar pulmonary sequestration, which is separated from the normal lung tissue by its own pleura, and intralobar pulmonary sequestration (ILS), which is an abnormal region within the normal lung parenchyma, without its own pleura. ILS which accounts for about 75% of the pulmonary sequestrations is located within a normal lobe and lacks its own visceral pleura. ILS is prone to pulmonary infection due to its connection to the tracheobronchial tree; thus, it is diagnosed at an early age (20 ± 8 years old) [8].

Bilateral ILS is extremely rare: there have been only 23 reported cases of bilateral ILS until now. ILS is supplied by an aberrant systemic artery which is usually from the descending thoracic aorta, followed by the abdominal aorta. An aberrant artery from the celiac artery and the left gastric artery as seen in our case is also extremely rare [[9], [10], [11]].

The treatment for ILS is surgical resection regardless of the symptoms, due to the potential risks of infection or hemoptysis. Over the decades, lobectomy through open thoracotomy has been traditionally performed to excise the sequestrated lung. For early stage non-small-cell lung cancer, VATS lobectomy is now accepted as an alternative surgical treatment to thoracotomy, because VATS is associated with lesser postoperative pain and better quality of life [12]. For the treatment of pulmonary sequestration, some retrospective studies have confirmed the feasibility of VATS, although the advantages of VATS over open thoracotomy remain controversial [[2], [3], [4],[13], [14], [15]]. Usually, the use of VATS for infections or for inflammatory conditions is uncommon, compared to its use for lung cancer [16]. Open thoracotomy continues to be the preferred choice for inflammatory conditions due to concerns about difficult visualization, bleeding, and an increased potential for injury due to the presence of adhesions. During resection of the ILS by the VATS approach, lobectomy is frequently performed because of the size, location, or inflammatory condition of the lesion. Limited resection such as segmentectomy, or wedge resection is avoided because of its technical complexity and the risk of uncomplete resection.

However, bilateral lobectomy (mostly bilateral lower lobectomy) through open thoracotomy for bilateral ILS might increase the risk for developing complications such as perioperative mortality and morbidity, decreased pulmonary function, respiratory muscle weakness, and stress on the heart. Therefore, a strategy to avoid bilateral lobectomy through open thoracotomy in necessary, because pulmonary sequestration is a benign disease and is detected in early life. Limited resection such as segmentectomy or wedge resection might be suitable if the sequestrated lesion is small and localized, without any inflammatory condition. In addition to preservation of the lung volume, minimal resection of the affected lesion using the VATS approach also has advantages such as cosmesis, less postoperative pain, and faster return to normal activities. Thus, we challenged the limited resection using a minimally invasive approach in the present case, since the patient was a young woman with bilateral ILS.

The right- sided ILS had a possibility of dense adhesions in the hilar structures and in the inferior mediastinum because the multiple cystic masses contained fluid and were complicated with infection. We decided to perform segmentectomy of the affected segments with VATS mini thoracotomy. However, the left-sided ILS had no apparent infection and matched with the Pryce Type 3: abnormal artery supplying only the sequestrated lung tissue. We were hopeful that the emphysematous change would help us to demarcate the resection line on the thoracoscopic image because the CT shows the sequestrated lesion as a hyperinflated area. In this case, the demarcated line was observed by referring to the telangiectasis of the sequestrated lung surface and the emphysematous changes. The resection line should be marked during the first thoracic procedure, because devascularization of the aberrant artery and lung collapse during lung ventilation might blur the planned resection line.

Nevertheless, VATS wedge resection of the sequestrated lung has a potential risk of difficulty in locating the boundary on thoracoscopy alone without the tactile sensation by the fingers, which can lead to an incomplete resection. The technique of visualizing the demarcation line using methylene blue dye or fluorescence imaging with indocyanine green during pulmonary resection has recently been reported [17,18]. These methods can help in identifying the resection line in cases of an undefined boundary.

## 4 Conclusions

It is important to plan the surgical treatment strategy for bilateral ILS for preservation of the respiratory function. Limited resection using the VATS approach for bilateral ILS is safe and can be used as a minimally invasive surgery.

## Consent

Written informed consent was obtained from the patient for publication of this case report and accompanying images.

## Authors' contributions

NI collected the data and drafted the manuscript. All authors participated in revison of the manuscript and made valuable contributions to the manuscript. All authors read and approved the final manuscript.

## Funding source

None.

## Ethical approval

We had an exemption from ethical approval because this was a case report, and not a trial or observational research.

## Provenance and peer review

Not commissioned, externally peer reviewed.

## Conflicts of interest

All the authors of the manuscript declare that they have no conflict of interest in connection with the presented manuscript.

## Registration of research studies

This article is a case report, and not a research study.

## Guarantor

Norichika Iga
